# Short-term effects of gastric bypass versus sleeve gastrectomy on high LDL cholesterol: The BASALTO randomized clinical trial

**DOI:** 10.1186/s12933-024-02296-x

**Published:** 2024-06-15

**Authors:** David Benaiges, Albert Goday, Anna Casajoana, Juana A. Flores-Le Roux, Montserrat Fitó, Oscar J. Pozo, Carme Serra, Manuel Pera, Gemma Llauradó, Elisenda Climent, Montserrat Villatoro, Iolanda Lazaro, Olga Castañer, Juan Pedro-Botet

**Affiliations:** 1 Department of Endocrinology and Nutrition, Hospital del Mar, Passeig Marítim, 25-29, Barcelona, 08003 Spain; 2 Department of Medicine, Universitat Pompeu Fabra, Plaça de la Mercè, 10-12, Barcelona, 08002 Spain; 3 Unit of Cardiovascular Risk and Nutrition, Institut Hospital del Mar d’Investigacions Mèdiques (IMIM), Dr. Aiguader, 80, Barcelona, 08003 Spain; 4 Consorci Sanitari de l’Alt Penedès i Garraf, Vilafranca del Penedès, Spain; 5 CiberOBN. Instituto de Salud Carlos III, Avenida Monforte de Lemos, 3-5. Pabellón 11. Planta 0, Madrid, 28029 Spain; 6 Department of Medicine, Universitat Autònoma de Barcelona. Plaça Cívica, Bellaterra, Barcelona, 08193 Spain; 7 Esophago-Gastric and Bariatric Surgery Unit, Department of Surgery, Hospital del Mar, Passeig Marítim, 25-29, Barcelona, 08003 Spain; 8 Applied Metabolomics Research Group, Neurosciences Research Program, IMIM (Hospital del Mar Research Institute), Dr. Aiguader 88, 08003 Barcelona, Spain; 9 Ciber Epidemiología y Salud Pública (CiberESP), Instituto de Salud Carlos III, Avenida Monforte de Lemos, 3-5. Pabellón 11. Planta 0, Madrid, 28029 Spain

**Keywords:** Roux-en-Y gastric bypass, Sleeve gastrectomy, LDL cholesterol, Hypercholesterolemia, Bariatric surgery, Lipoprotein, Cholesterol esters

## Abstract

**Background:**

There has been a substantial increase in the use of laparoscopic sleeve gastrectomy (SG) to treat morbid obesity despite observational evidence demonstrating the superiority of Roux-en-Y gastric bypass (RYGB) for reducing low-density lipoprotein (LDL) cholesterol. The main aim was to ascertain whether high LDL cholesterol levels should be considered when selecting the most appropriate surgical procedure for each patient (RYGB or SG).

**Methods:**

In this single-center, randomized clinical trial using intention-to-treat analysis, 38 patients with severe obesity and elevated levels of LDL cholesterol were randomly assigned to undergo RYGB or SG. The primary outcome was LDL cholesterol remission at 12 months, defined as LDL cholesterol < 3.36 nmol/l without lipid-lowering medications. Secondary outcomes included changes in weight, other comorbidities, qualitative lipoprotein traits, cholesterol esters, glycoproteins, cholesterol absorption and synthesis metabolites and complications.

**Results:**

Intention-to-treat analysis revealed that LDL cholesterol remission occurred in 66.6% of RYGB patients compared to 27.8% of SG patients (*p* = 0.019). Among patients completing follow-up, RYGB demonstrated superior remission (80.0% vs. 29.4%, *p* = 0.005). Exclusive benefits of RYGB included a reduction in large, medium, and small LDL particles. Cholesterol absorption markers showed differential behavior after both techniques: campesterol (Δ −15.2 µg/mg, 95% CI −30.2 to −0.1) decreased after RYGB, and sitosterol (Δ 21.1 µg/mg, 95% CI 0.9 to 41.2), cholestanol (Δ 30.6 µg/mg, 95% CI 14.8 to 57.9) and campesterol (Δ 18.4 µg/mg, 95% CI 4.4 to 32.3) increased after SG. No differences in weight loss, cholesterol esters, glycoproteins, cholesterol synthesis metabolites or postoperative complications were observed between techniques.

**Conclusion:**

In conclusion, RYGB is superior to SG in terms of short-term of high LDL cholesterol remission. Furthermore, RYGB also led to a greater improvement in lipoprotein parameters that confer an atherogenic profile. Therefore, the presence of elevated levels of LDL cholesterol should be considered when determining the optimal bariatric surgery procedure for each patient.

**Trial registration:**

Clinicaltrials.gov number, NCT03975478).

**Graphical abstract:**

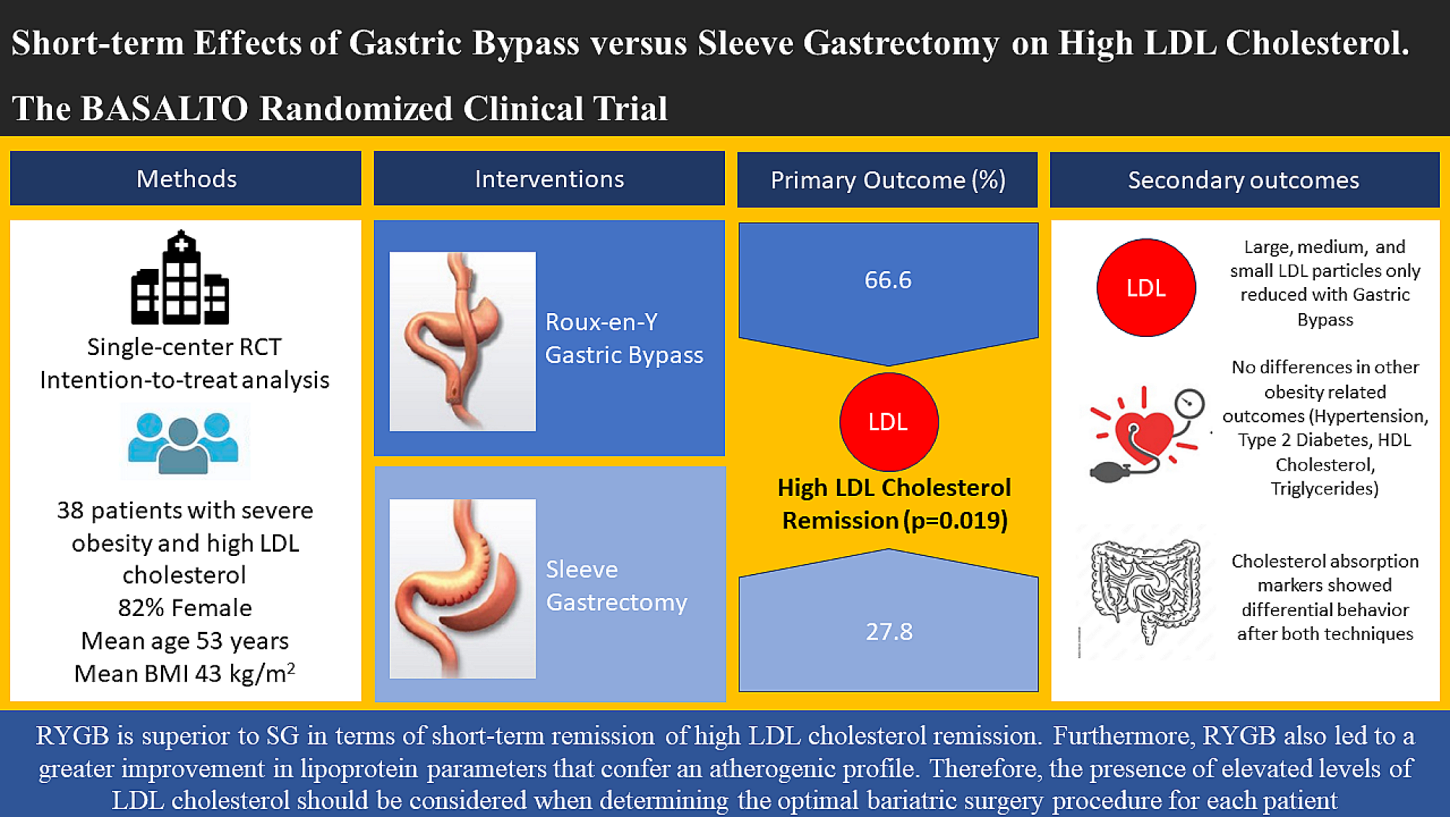

**Supplementary Information:**

The online version contains supplementary material available at 10.1186/s12933-024-02296-x.

## Introduction

Observational studies and randomized controlled trials (RCTs) have consistently demonstrated the superiority of bariatric surgery over conventional treatment in terms of achieving substantial weight loss and ameliorating related obesity complications such as diabetes, hypertension, and dyslipidemia [1–4]. This evidence has led to a shift toward personalized medicine to treat severe obesity. In this regard, one of the current challenges in bariatric surgery is the selection of the optimal bariatric surgery technique.

In the early 21st century, sleeve gastrectomy (SG) gained popularity because of its favorable short-term outcomes, increased technical simplicity and safety profile. Over the past decade, the use of SG has increased markedly; the number of SGs surpassed that of RYGBs in 2018, and in 2023, SG was performed more than twice as frequently as RYGB (63.3% vs. 28.8% of all surgeries worldwide) [5, 6]. 

In this context the pros and cons of both techniques play a pivotal role in the clinical decision-making process for determining the most appropriate bariatric surgery procedure [7, 8]. To date, there have been few RCTs comparing both surgical techniques, and they have primarily focused on weight loss or type 2 diabetes remission [9–11]. In this context, some experts have emphasized the need for RCTs that specifically target lipid alterations and investigate the underlying mechanisms driving these changes [12]. This is supported for different reasons. First, different observational studies and meta-analyses have reported the superiority of RYGB in terms of improving LDL cholesterol concentrations, with no differences in other lipoproteins [13, 14]. Second, LDL cholesterol is the main driver and causal factor of atherosclerotic cardiovascular disease [15]. Third, dyslipidemia is one of the most prevalent obesity complication found in patients with severe obesity undergoing bariatric surgery in the majority of cohorts [1]. 

Furthermore, in certain clinical scenarios that involve an insulin resistance state, such as diabetes mellitus, obesity or metabolic syndrome, the measurement of LDL cholesterol is insufficient to evaluate the cardiovascular risk. In such cases, 2D nuclear magnetic resonance (NMR) spectrometry provides a direct analysis of lipid metabolism beyond the usual clinical parameters since it differentiates the properties of different lipoproteins in their respective subfractions, characterizes them in terms of their composition and size, and quantifies the number of each of these particles [16]. 

Based on all the aforementioned factors, we designed the first RCT with an intention-to-treat analysis of patients with severe obesity to ascertain before bariatric surgery whether high LDL cholesterol levels should be considered when selecting the most appropriate surgical procedure for each patient (RYGB or SG). The primary objective was to compare LDL cholesterol remission at 12 months after RYGB and SG. The secondary aims of this trial were to examine the evolution of other obesity complications, weight, complications, conventional lipid profile, lipoprotein particle composition, glycoproteins, selected cholesterol esters and markers of cholesterol absorption and synthesis.

## Methods

### Study design

The study design, rationale and methods, including operative techniques, have been published previously [17]. This is a phase 3, single-center, RCT with an intention-to-treat analysis involving patients randomized to undergo either laparoscopic SG or RYGB from November 2019 to February 2022, with a 1-year follow-up after surgery.

The study was conducted in accordance with the principles of the Declaration of Helsinki, approved by a local ethical committee (2019/8471/I), and registered at clinicaltrials.gov (NCT03975478). All patients gave written informed consent. The findings were reported in line with the Consolidated Standards of Reporting Trials (CONSORT) guidelines [18]. 

### Participants

The inclusion criteria were as follows: body mass index (BMI) ≥ 40 or ≥ 35 kg/m^2^ with significant obesity-related complications, age 18–60 years, previous unsuccessfully instituted and supervised diet and exercise program, elevated levels of LDL cholesterol [> 3.36 nmol/l (130 mg/dl) or under lipid-lowering drug].

The overall exclusion criteria were as follows: BMI > 60 kg/m^2^ and previous bariatric surgery. Additional exclusion criteria for undergoing bariatric surgery were as follows: significant psychiatric disorder, severe eating disorder, active alcohol or substance abuse, contraindications for major abdominal surgery, active gastric ulcer disease, severe liver disease, pregnancy or breastfeeding. Also excluded were instances where SG or RYGB were preferred in cases of severe symptomatic gastroesophageal reflux disease despite medication, large hiatal hernia, expected presence of dense adhesions at the small bowel level, need for endoscopic follow-up of the duodenum, history of inflammatory bowel disease, and history of kidney transplantation in which drug malabsorption can be caused by a RYGB. Finally, perioperative statin cannot be withdrawn in the following scenarios, resulting in their exclusion: established or subclinical cardiovascular disease, LDL cholesterol > 4.91 nmol/l or a history of familial hypercholesterolemia [17]. 

### Randomization

Subjects were randomized (1:1) to undergo either RYGB or SG. A central, computer-based block randomization with sealed envelopes was carried out. There was no blinding regarding the type of surgery; therefore, patients as well as physicians and dietitians assessing follow-up data were informed of the performed procedure. The researcher responsible for statistical analysis was blinded to group allocation until data analysis was completed.

### Cholesterol-lowering treatment management

Management of cholesterol-lowering treatment was based on local clinical practice guidelines [19]. Cholesterol-lowering treatment was started in primary prevention in adults with LDL cholesterol > 4.91 nmol/l (> 190 mg/dl) or > 3.36 nmol/l (> 130 mg/dl) with a 10-year estimated cardiovascular risk > 10% using REGICOR adaptation of the Framingham Risk Score [20]. Similarly, cholesterol-lowering therapy was withdrawn in patients with a 10-year cardiovascular risk < 10% and with no previous LDL cholesterol determinations > 4.91 nmol/l. To avoid bias that may arise due to an open study, a standardized protocol was followed for the two groups regarding dietary recommendations and physical activity. Preoperatively, treatment was assessed before randomization, and postoperatively, assessment occurred at the 3-, 6- and 12-month follow-up visits.

### Outcome measures

Patients were evaluated by a multidisciplinary team prior to and at 3, 6 and 12 months after bariatric surgery.

The primary outcome was 12 months LDL cholesterol remission defined by postoperative LDL cholesterol < 3.36 nmol/l (< 130 mg/dl) without cholesterol-lowering drugs. The secondary outcomes included other obesity complications, weight, conventional lipid profile, characterization of lipoproteins, glycoproteins, selected cholesterol esters and absorption and synthesis cholesterol metabolites. Study outcomes were measured at each visit.

For the obesity complications evaluated, LDL cholesterol improvement was defined as a decrease ≥ 20% in LDL cholesterol levels without cholesterol-lowering drugs in patients without preoperative lipid-lowering treatment. In patients on lipid-lowering treatment before surgery, LDL cholesterol improvement was defined as medication withdrawal and LDL cholesterol < 3.36 nmol/l or decrease ≥ 20% in LDL cholesterol concentration without medication withdrawal. Hypertriglyceridemia remission was considered when triglycerides were < 1.69 nmol/l without fibrate treatment. Low HDL cholesterol remission was defined as HDL cholesterol > 1.29 nmol/l in women or > 1.03 nmol/l in men. Diabetes remission was defined according to American Diabetes Association criteria for complete remission: glycated hemoglobin [HbA1c] value < 6.0% (42 mmol/mol) and fasting glucose level < 5.6 mmol/l without diabetes medication [21]. Metabolic syndrome was assessed based on the criteria of the International Diabetes Federation [22]. 

Targeted lipidomic approaches developed at the Applied Metabolomics Research Group at Hospital del Mar Research Institute were applied to provide further insights into the mechanism(s) responsible for the bariatric surgery-induced lowering effects on LDL cholesterol, including changes in precursors and metabolites of cholesterol and cholesteryl esters.

The measurement of cholesterol precursors and metabolites was performed by gas chromatography‒mass spectrometry (GC‒MS). The analytical method was adapted from previous works in the literature [23–25]. 

We additionally determined the effects of the intervention on the five most abundant cholesteryl esters in plasma [26]. To this end, forty microliters of plasma were spiked with 16.3 µg of the internal standard (ISTD) cholesteryl nonadecanoate (Avanti, Merck), and the lipids were extracted with 2 mL of chloroform/methanol (2:1 v/v). After centrifugation (5 min at 3,500 rpm), the organic phase was transferred to a new borosilicate glass tube and evaporated to dryness under N_2_ at 30 °C. Cholesteryl esters were isolated by solid-phase extraction and trans-esterified using acidified methanol to prepare fatty acid methyl esters (FAMEs), as previously described [27]. FAMEs were injected into the GC/MSEI and separated with a J&W DB-FastFAME capillary column (30 m × 0.2 mm × 0.25 μm film thickness, Agilent). The injector temperature was set at 250 °C, and 1-µL injections were performed (split ratio 15:1). GC was run using an optimized temperature program as follows: the program started at 125 °C, increased to 230 °C at a rate of 25 °C/min, held for 1 min, increased to 245 °C at a rate of 4 °C/min, and held for 7 min. Helium was used as a carrier gas (14 psi, constant pressure mode). FAMEs were detected using the selected ion monitoring (SIM) mode. Several m/z ions common to saturated, monounsaturated, and polyunsaturated FAMEs were monitored [28]. 

Characterization of the different lipoproteins was performed by 2D NMR spectrometry (LIPOSCALE TEST®). This method can be used to determine cholesterol and triglyceride contents of the main lipoprotein fractions (VLDL, IDL, LDL and HDL) in plasma. It can also be used to determine the particle concentration (total, large, medium and small) of each main fraction (VLDL, LDL and HDL) and the size of the main lipoprotein fractions (VLDL, LDL and HDL) [29]. 

All subjects completed a dietary record for three consecutive days from Sunday to Tuesday. Baseline nutrient intake was calculated with DIET ANALYSIS NUTRITIONIST IV software (N Squared Computing, San Bruno, CA, USA). Physical activity was measured using the REGICOR Short Physical Questionnaire [30]. 

### Statistical analysis

The power estimation was based on the assumption of an LDL cholesterol remission rate of 70% in the RYGB group and 20% in the SG. A sample size of 18 patients per arm was estimated to provide 80% power to detect a difference between groups using a two-sided α of 0.05. These estimates were derived from previously reported data from our group [13]. A drop-out rate of 20% was anticipated.

All data were collected in a central database according to a standardized protocol. Data are expressed as the mean ± standard deviation for continuous variables after a normal distribution, as the median with interquartile range for continuous variables with a nonnormal distribution and as percentages and frequencies for categorical variables. The normality of the models was evaluated visually and by the Kolmogorov‒Smirnov test. For skewed variables, a logarithmic transformation was used to achieve normality. Student’s t test was performed to assess differences between 2 means. Chi-square or Fisher’s exact tests were used to assess the degree of association among categorical variables (including the primary outcome: LDL cholesterol remission at 1 year). ANOVA models were used to analyze the evolution of continuous variables in each group and assess differences between the groups at each time point from baseline. Furthermore, statistical adjustments were performed to control for baseline characteristics that significantly differed between the groups. To correct for multiple comparisons, the Bonferroni adjustment was applied. Patients under cholesterol-lowering treatment at baseline or at the 12-month follow-up were excluded from the analysis of lipid level changes after bariatric surgery. Data imputation was performed for patients who did not attend the 3- or 6-month follow-up visits. A two-sided p value < 0.05 was considered statistically significant. Statistical analysis was performed with SPSS (version 28.0 for Windows; SPSS, Chicago, IL, USA).

## Results

**Fig. 1:**
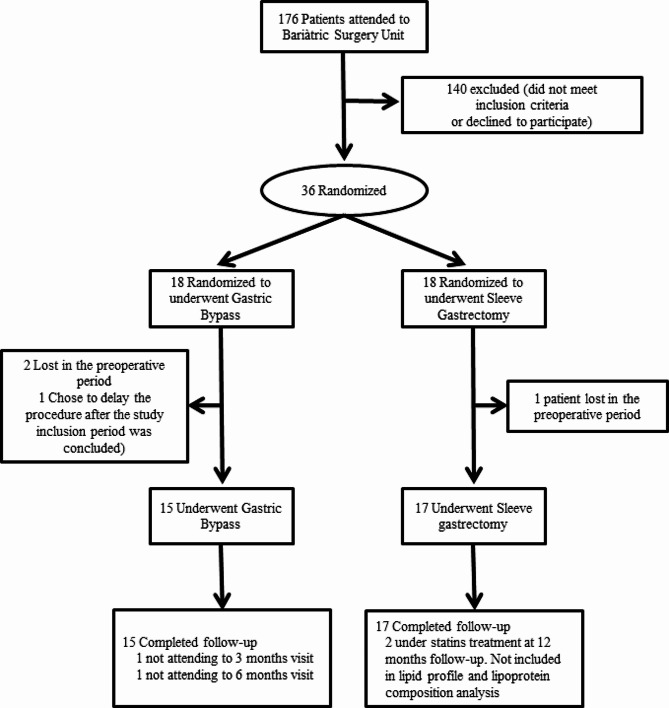
Study flow chart

Figure 1 shows the flow of participants through the trial. Seventeen patients in the SG group and 15 in the RYGB group underwent surgery and completed a one-year follow-up, with a global adherence rate of 88.9%.

Statins were withdrawn following the study protocol in 9 of the 10 patients under statin treatment at inclusion. One patient continued statin because his estimated 10-year cardiovascular risk was > 10%; he was randomized to SG. One patient in the SG group restarted statins at the three-month follow-up because her LDL cholesterol was > 4.91 nmol/l. In two patients in the SG group, statin therapy was prescribed at the 3- or 6-month follow-up by general practitioners; in both cases, statins were withdrawn, and at the 12-month follow-up, the primary outcome was assessed.

### Baseline characteristics

The baseline characteristics of the study population are shown in Table 1. No differences between groups were observed except for triglyceride levels, which were higher in the SG group.

**Table 1:** Baseline characteristics of the patients included in the study

	RYGB (*n* = 18)	SG (*n* = 18)	*P*
Sex, n (% females)	16 (88.9)	15 (83.3)	0.500
Age (years), median (IQ range)	50.5 (44.0-56.3)	54.5 (50.3–57.3)	0.136
Weight (kg), mean ± SD	118.6 ± 15.3	109.33 ± 12.7	0.055
BMI (kg/m^2^), mean ± SD	43.5 ± 3.6	43.2 ± 4.1	0.837
Abdominal circumference (cm), median (IQ range)	128 (122.3–137.0)	121.3 (115.5–131.0)	0.113
Daily caloric intake (Kcal/day), mean ± SD	1592 ± 427	1694 ± 613	0.643
Physical activity (METs), median (IQ range)	1624 (423–2399)	1657 (1254–2967)	0.233
Smoking habit, n (%)	5 (27.8)	4 (22.2)	0.500
Statin therapy at inclusion, n (%)	3 (16.7)	7 (38.9)	0.132
Statin therapy before randomization, n (%)	0 (0)	1 (5.6)	0.310
Hypertriglyceridemia, n (%)	9 (50)	13 (72.2)	0.153
Fibrate therapy, n (%)	1 (5.6)	1 (5.6)	0.757
Low HDL cholesterol, n (%)	9 (50)	13 (72.2)	0.153
Type 2 diabetes, n (%)	5 (27.8)	6 (33.3)	0.500
Hypertension, n (%)	9 (50.0)	11 (61.1)	0.369
Metabolic Syndrome, n (%)	14 (77.8)	17 (94.4)	0.169
Glucose (mmol/L), median (IQ range)	5.8 (5.2–6.4)	5.9 (5.2–6.6)	0.728
Total cholesterol (mmol/L), mean ± SD	5.66 ± 0.61	5.8 ± 0.67	0.233
LDL cholesterol (mmol/L), mean ± SD	3.86 ± 0.47	3.92 ± 0.55	0.726
HDL cholesterol (mmol/L), mean ± SD	1.32 ± 0.3	1.32 ± 0.3	0.329
Triglycerides (mmol/L), mean ± SD	1.62 ± 0.53	2.23 ± 0.8	0.018
Lp(a) (nmol/L), median (IQ range)	56.0 (31.7-145.1)	37.2 (10.9-116.3)	0.241

BMI, body mass index; LDL, low-density lipoprotein; METs, metabolic equivalents; HDL, high-density lipoprotein; IQ, interquartile; Lp(a), lipoprotein(a); RYGB, Roux-en-Y gastric bypass; SG, sleeve gastrectomy

### Primary outcomes

**Fig. 2:**
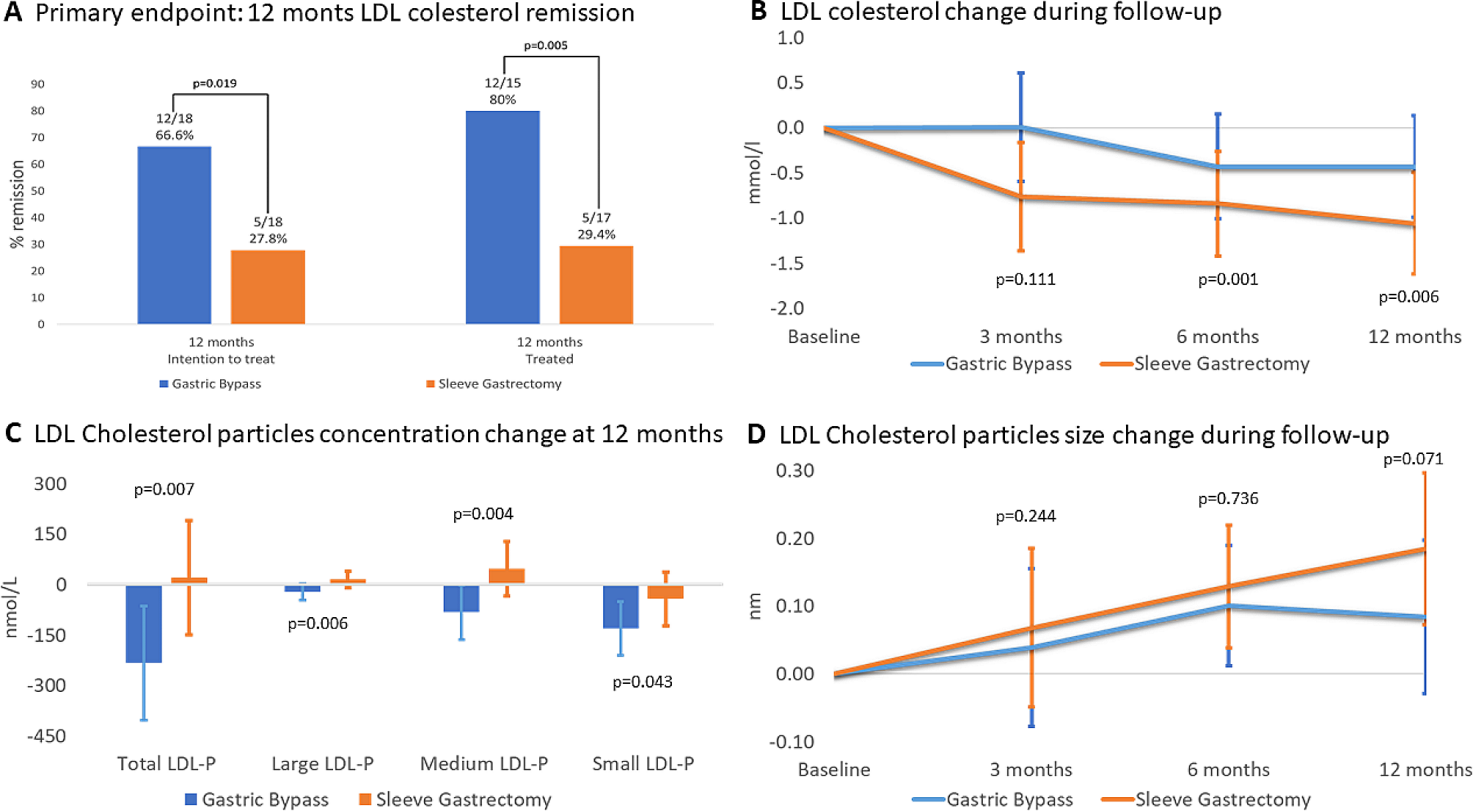
Primary endpoint (LDL cholesterol remission) and secondary outcomes related to LDL cholesterol. The differences in high LDL cholesterol remission (Fig. 2A) were determined using a chi-square test. Figure 2B and D show changes observed during follow-up. Negative values indicate a reduction, whereas positive values indicate an increase.  Data are expressed as the means with 95% confidence intervals. The p value refers to the comparison between groups at each time interval. The changes in these parameters were analyzed using repeated-measures ANOVA ( p  < 0.05) adjusted for baseline triglycerides.  LDL, low-density lipoprotein

LDL cholesterol remission was observed at 12 months in 12 of 18 (66.6%) patients in the RYGB group and in 5 of 18 (27.8%) patients in the SG group (*p* = 0.019). When the analysis included only those patients who underwent bariatric surgery and who completed follow-up, LDL cholesterol remission was observed in 80.0% of patients in the RYGB group and 29.4% of patients in the SG group (*p* = 0.005) (Fig. 2A).

### Secondary outcomes

#### Otherlipid disturbances and obesitycomplications

LDL cholesterol remission at 3 and 6 months and 12 months LDL cholesterol improvement were greater in the RYGB group. Additionally, a higher remission of LDL at 12 months was observed in non-diabetic patients. No differences between groups were observed regarding other lipid disturbances or obesity complications remission (Table 2).

**Table 2:** Comorbidity outcomes of the study groups

	RYGB	SG	*P*
3-months LDL cholesterol remission, n (%)	10/12 (83.3)	4/17 (23.5)	0.002
6-months LDL cholesterol remission, n (%)	12/14 (85.7)	3/17 (17.6)	< 0.001
12-months LDL cholesterol improvement, n (%)	12/15 (80.0)	5/17 (29.4)	0.005
12-months LDL cholesterol remission in patients with type 2 diabetes, n (%)	4/4 (100.0)	2/6 (33.3)	0.071
12-months LDL cholesterol remission in patients without type 2 diabetes, n (%)	8/11 (72.3)	3/11 (27.3)	0.043
12-months hypertriglyceridemia remission, n (%)	6/8 (75.0)	11/12 (91.7)	0.344
12-months low HDL cholesterol remission, n (%)	7/8 (87.5)	9/12 (75)	0.465
12-months type 2 diabetes remission, n (%)	2/4 (50)	3/6 (50)	0.783
12-months hypertension remission, n (%)	5/8 (62.5)	6/10 (60)	0.648
12-months Metabolic Syndrome remission, n (%)	14/14 (100)	14/17 (82.4)	0.151

Outcomes were analyzed in patients who presented the comorbidity at baseline before surgery and who completed follow-up. LDL, low-density lipoprotein; HDL, high-density lipoprotein; RYGB, Roux-en-Y gastric bypass; SG, sleeve gastrectomy

#### Anthropometric outcomes

**Fig. 3:**
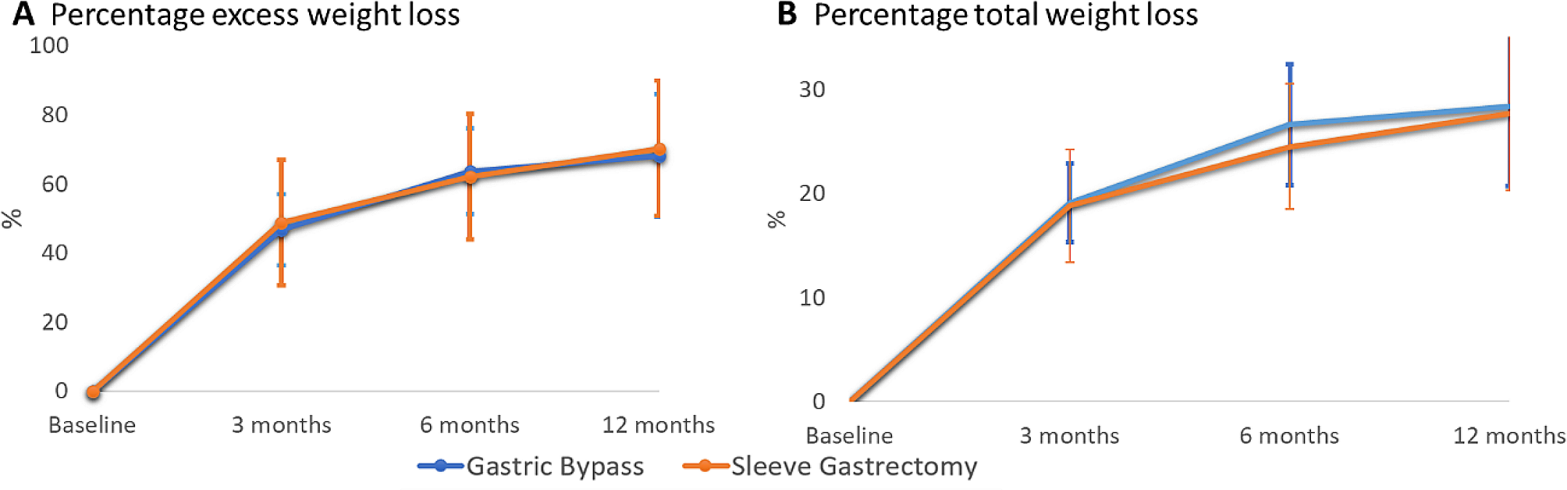
Changes in the percentage of excess weight loss (**A**) and percentage total weight loss (**B**). Data are expressed as the means ± standard deviations. Significance at *p* < 0.05 was determined using repeated-measures ANOVA

No differences were found in percentage excess weight loss and percentage total weight loss between groups (Fig. 3).

#### Biochemical parameter outcomes

Changes in biochemical parameters after intervention were assessed in the 15 SG and 15 RYGB patients who completed follow-up and were not taking cholesterol-lowering drugs at baseline or at the 12-month follow-up. The biochemical parameter changes at the 12-month follow-up for each technique and the differences between both are detailed in Table 3. Short-term changes in these parameters at the 3- and 6-month follow-ups are presented in the supplementary Figs. 1–7. Both groups were comparable at baseline, except for higher medium LDL particles in the SG group.

**Table 3:** Biochemical parameters at baseline and 12 months after gastric bypass and sleeve gastrectomy

	Roux-en-Y Gastric Bypass (*n* = 15)			Sleeve Gastrectomy (*n* = 15)			Between groups difference	
	Baseline	12 months	Difference, baseline to 12 months	Baseline	12 months	Difference, baseline to 12 months	Crude p value	Adjusted p value
*Conventional lipid profile (mmol/L)*								
Total cholesterol	5.72 ± 0.62	4.98 ± 0.96	−0.74 ( −1.48 to 0.01)	5.58 ± 0.47	5.63 ± 0.94	0.04 ( −0.69 to 0.77)	0.043	0.015
LDL cholesterol	3.90 ± 0.50	3.02 ± 0.50	−0.88 ( −1.45 to −0.31)*	3.74 ± 0.35	3.71 ± 0.84	−0.03 ( −0.61 to 0.54)	0.006	0.018
HDL cholesterol	1.31 ± 0.30	1.55 ± 0.25	0.24 (0.06 to 0.42)*	1.18 ± 0.21	1.49 ± 0.27	0.31 (0.13 to 0.49)*	0.400	0.561
Triglycerides	1.67 ± 0.53	1.24 ± 0.45	−0.43 ( −0.99 to 0.13)	2.18 ± 0.90	1.11 ± 0.39	−1.05 ( −1.62 to −0.50)*	0.014	0.254
Lipoprotein(a)	103.1 ± 69.5	109.1 ± 83.7	6.1 ( −60.4 to 72.5)	78.7 ± 100.7	82.7 ± 104.5	− 0.0 ( −57.5 to 65.6)	0.949	0.949
*Nuclear magnetic resonance subclasses*								
VLDL-P number (nmol/L)								
Total	59.81 ± 21.05	45.70 ± 16.33	−21.36 ( −34.7 to −8.03)*	76.19 ± 30.2	41.45 ± 13.97	−27.50 ( −40.8 to −14.17)*	0.049	0.376
Large	1.41 ± 0.44	1.15 ± 0.33	−0.39 ( −0.65 to −0.39)*	1.76 ± 0.56	1.01 ± 0.27	−0.62 ( −0.88 to −0.36)*	0.013	0.091
Medium	7.48 ± 3.23	5.73 ± 1.83	−2.94-5.29 to −0.59)*	9.99 ± 5.11	5.38 ± 2.23	−3.42 ( −5.76 to −1.07)*	0.103	0.694
Small	50.93 ± 17.48	38.81 ± 14.31	−18.04 ( −29.20 to −6.87)*	64.44 ± 24.82	35.06 ± 11.74	−23.46 ( −34.62 to −12.30)*	0.046	0.350
*VLDL-P composition (mmol/L)*								
VLDL-C	0.53 ± 0.18	0.39 ± 0.18	−0.14 ( −0.34 to 0.06)	0.71 ± 0.31	0.38 ± 0.16	−0.32 ( −0.15 to −0.49)*	0.046	0.619
VLDL-TG	0.94 ± 0.36	0.72 ± 0.24	−0.34 (−0.55 to −0.12)*	1.2 ± 0.49	0.64 ± 0.22	−0.44 ( −0.66 to −0.26)*	0.044	0.411
VLDL size (nm)	15.8 ± 2.5	16.4 ± 2.8	0.06 ( −0.10 to 0.21)	15.2 ± 3.0	15.8 ± 2.4	0.06 ( −0.10 to 0.22)	0.752	0.091
*LDL-P number (nmol/L)*								
Total	1432.02 ± 198.81	1198.4 ± 155.1	−233.31 ( −403.11 to −63.52)*	1319.29 ± 90.85	1339.7 ± 200.1	20.09 ( −149.7 to 189.88)	0.004	0.007
Large	227.37 ± 30.97	205.7 ± 25.5	−21.34 ( −45.4 to 2.75)	213.13 ± 15.29	229.0 ± 26.6	15.51 (-8.58 to 39.60)	0.003	0.006
Medium	435.01 ± 89.56	355.3 ± 80.5	−82.11 ( −162.7 to −1.57)*	370.34 ± 48.24	414.9 ± 93.9	47.01 ( −33.53 to 127.55)	0.003	0.004
Small	769.65 ± 92.67	637.4 ± 76.2	−129.87 ( −210.51 to −49.23)*	735.83 ± 54.81	695.7 ± 88.9	−42.43 ( −123.1 to 38.21)	0.023	0.043
*LDL-P composition (mmol/L)*								
LDL-TG	0.20 ± 0.03	0.16 ± 0.05	−0.04 ( −0.08 to −0.01)*	0.20 ± 0.04	0.18 ± 0.04	−0.01 ( −0.04 to 0.03)	0.016	0.065
LDL size (nm)	21.11 ± 0.13	21.2 ± 0.19	0.084 ( −0.029 to 0.197)	21.05 ± 0.12	21.23 ± 0.09	0.185 (0.073 to 0.297)*	0.274	0.071
*HDL-P number (nmol/L)*								
Total	26.63 ± 2.9	26.13 ± 3.08	1.10 ( −1.87 to −.06)	26.12 ± 3.45	25.50 ± 3.06	1.41 ( −1.55 to 4.37)	0.658	0.835
Large	0.31 ± 0.03	0.31 ± 0.04	−0.01 ( −0.43 to 0.02)	0.3 ± 0.04	0.31 ± 0.03	0.01 ( −0.02 to 0.04)	0.575	0.131
Medium	10.49 ± 0.96	11.29 ± 1.32	0.70 (0.06 to 1.35)*	10.64 ± 1.39	10.82 ± 1.03	0.62 ( −0.02 to 1.26)	0.132	0.800
Small	15.82 ± 2.46	14.53 ± 2.78	0.41 ( −2.19 to 3.00)	15.17 ± 3.04	14.37 ± 2.39	0.79 ( −1.81 to 3.38)	0.991	0.777
*HDL-P composition (mmol/L)*								
HDL-TG	0.16 ± 0.03	0.16 ± 0.05	−0.01 ( −0.04 to −0.03)	0.18 ± 0.06	0.14 ± 0.04	−0.02 ( −0.06 to 0.01)	0.063	0.391
HDL size (nm)	8.33 ± 0.05	8.34 ± 0.08	0.01 ( −0.039 to 0.059)	8.34 ± 0.07	8.34 ± 0.05	−0.001 ( −0.05 to 0.05)	0.399	0.133
*Glycoproteins (µmol/L)*								
Glycoprotein-A	786.7 ± 96.2	649.0 ± 93.8	−169.0 ( −244.4 to −93.5)*	865.0 ± 130.1	658.0 ± 91.2	−169.0 ( −244.4 to −93.5)*	0.169	0.324
Glycoprotein -B	369.2 ± 42.8	318.8 ± 26.9	−58.9 ( −87.5 to −30.4)*	389.6 ± 49.9	327.2 ± 48.8	−53.9 ( −82.5 to −25.4)*	0.471	0.734
Glycoprotein -F	241.4 ± 51.8	214.9 ± 34.4	−45.3 ( −76.1 to −14.5)*	291.5 ± 67.9	211.5 ± 25.7	−61.1 ( −91.9 to −30.3)*	0.041	0.846
*Cholesteryl esters (proportion of quantified fatty acids)*								
C16:0	12.9 ± 1.8	13.3 ± 1.4	0.28 ( −0.73 to 1.29)	13.2 ± 1.3	12.8 ± 1.3	−0.32 ( −1.33 to 0.69)	0.097	0.253
C16:1n-7	2.6 ± 1.2	2.3 ± 1.1	−0.26 ( −1.19 to 0.66)	3.1 ± 1.2	2.0 ± 1.1	−1.07 ( −1.99 to −0.15)*	0.071	0.099
C18:1n-9cis	15.6 ± 3.2	16.9 ± 4.4	0.93 ( −1.60 to 3.46)	17.0 ± 3.0	17.7 ± 3.8	1.07 ( −1.46 to 3.60)	0.629	0.915
C18:2n-6cis	52.9 ± 7.0	51.4 ± 6.1	−1.02 ( −5.21 to 3.17)	50.4 ± 4.9	51.8 ± 6.3	0.88 ( −3.31 to 5.08)	0.165	0.381
C20:4-n6	7.2 ± 1.4	7.4 ± 1.3	0.17 ( −0.80 to 1.14)	7.7 ± 1.6	7.3 ± 2.3	−0.36 ( −1.33 to 0.61)	0.616	0.297
*Cholesterol absorption biomarkers (µg/mg)*								
Sitosterol	47.0 ± 26.5	26.4 ± 10.0	−20.6 ( −42.3 to 1.0)	51.4 ± 17.9	72.4 ± 31.1	21.1 (0.9 to 41.2)*	< 0.001	< 0.001
Cholestanol	81.3 ± 23.4	81.9 ± 19.3	0.6 ( −20.9 to 22.1)	82.4 ± 21.0	118.7 ± 38.0	36.3 (14.8 to 57.9)*	0.002	0.001
Campesterol	31.5 ± 16.9	16.4 ± 8.5	−15.2 ( −30.2 to −0.1)*	31.2 ± 10.5	49.5 ± 25.3	18.4 (4.4 to 32.3)*	0.001	< 0.001
*Cholesterol synthesis biomarkers (µg/mg)*								
Desmosterol	72.5 ± 80.9	55.8 ± 57.7	−16.7 ( −32.5 to −0.9)*	57.7 ± 19.4	40.1 ± 16.3	−21.1 ( −42.1 to −0.1)*	0.405	0.460
Lathosterol	81.3 ± 36.9	60.2 ± 35.0	−17.5 ( −33.3 to −1.8)*	72.8 ± 21.2	43.5 ± 26.6	−29.3 ( −50.3 to −8.3)*	0.505	0.763

Baseline and 12-month values were expressed as the means ± standard deviations. Within-group changes at the 12-month follow-up were expressed as the means with 95% confidence intervals. ANOVA models with Bonferroni adjustment were used to analyze the evolution of continuous variables in each group and assess differences between the groups from baseline. Differences between groups were expressed crude and adjusted for baseline triglycerides

*Within-group significant change during follow-up (*p* < 0.05)

HDL, high-density lipoprotein; LDL, low-density lipoprotein

#### Conventional lipid profile outcomes

The two surgical techniques that were assessed showed a differential impact on LDL cholesterol levels during follow-up (Fig. 2B): there was an early decrease after RYGB, whereas LDL cholesterol levels remained stable after SG. Similar changes were observed in total cholesterol. Triglycerides displayed a progressive decline during follow-up. The triglyceride reduction was more pronounced in the SG group; however, it became nonsignificant after adjusting for baseline triglycerides. HDL cholesterol presented an initial decrease at 3 months only after RYGB, and it increased progressively thereafter in both groups, with 12-month values higher than baseline. There were no differences in lipoprotein(a) changes between the two groups.

#### Lipoprotein composition outcomes

A decrease in total, small, medium and large LDL particles were observed after RYGB with a neutral effect after SG (Fig. 2C). Significant differences between techniques were already detected at the 6-month follow-up (Supplemental Fig. 2). LDL particle size increased after both techniques without differences between them; this rise was significant at the 6- and 12-month follow-ups with SG and at the 6-month follow-up with RYGB (Fig. 2D). Only RYGB was associated with a reduction in the TG content of LDL; however, no differences between groups were detected.

No differences in VLDL changes between techniques were observed after adjusting for baseline triglycerides. A significant decrease in cholesterol and triglyceride content in VLDL with both techniques was detected at the 3-month follow-up. Moreover, a reduction in total, small, medium and large VLDL particles was detected at 3 months after SG and at 6 months after RYGB (supplemental Fig. 3). No changes in VLDL size were found.

There were no differences in HDL triglycerides and HDL particle changes between the two techniques. Both techniques led to a transient reduction in HDL particles at 3 months, primarily driven by a decrease in small HDL particles (supplemental Fig. 4). Both techniques increased 12-month medium HDL cholesterol particles; however, the increase was nonsignificant in the SG group (*p* = 0.064). HDL particle size increased only at 3 months in both groups.

#### Glycoprotein outcomes

Glycoproteins A, B, and F showed a similar progression after both procedures: a significant decrease was observed at 3 months and was sustained throughout follow-up.

#### Cholesterol ester outcomes

Chol C16:1n7 significantly decreased only after SG; the differences between the techniques did not achieve significance. For the remaining selected cholesterol esters, no differences in their levels were detected, either within or between techniques.

#### Metabolite outcomes

**Fig. 4:**
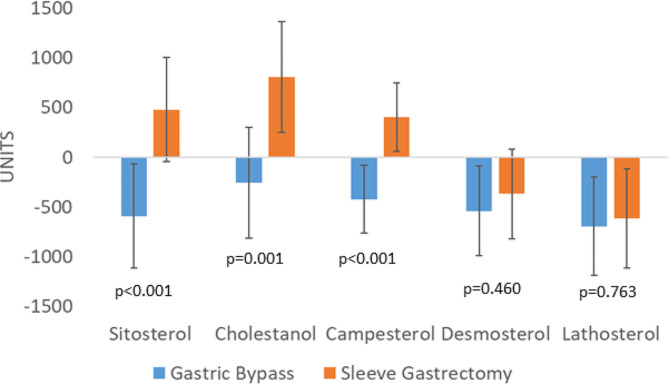
Changes in cholesterol absorption and synthesis markers with RYGB and SG

Changes in the three metabolites of intestinal absorption were clearly different between the techniques. They decreased after RYGB, reaching statistical significance for campesterol. In contrast, all markers of intestinal cholesterol absorption increased after SG. Furthermore, markers of synthesis decreased after both techniques, and there were no differences between them (Fig. 4). Most of these changes were already evident 3 months after the surgery (Supplemental Fig. 7).

#### Complications

Two patients presented surgical complications, and both underwent RYGB. One patient experienced extraluminal bleeding necessitating a transfusion of 2 units of red blood cells, and the other developed renal insufficiency requiring intravenous fluid therapy. No mortality was observed.

### Discussion

The present RCT demonstrated the short-term superiority of RYGB compared to SG in terms of achieving remission of elevated levels of LDL cholesterol in severely obese patients. Additionally, the BASALTO study provided new data on qualitative lipoprotein changes after surgery that may improve the atherogenic profile and suggested possible mechanisms associated with these changes.

These results provide further support for the superiority of RYGB over SG in terms of LDL cholesterol [31–36]. For instance, a meta-analysis conducted by our group observed that hypercholesterolemia remission was more frequent at one year after RYGB than after SG (RR: 1.43, 95% CI: 1.27 to 1.61), and the reduction in LDL cholesterol levels was greater after RYGB than after SG (mean difference: 19.29 mg/dL, 95% CI: 11.93 to 26.64) [14]. 

To date, beyond gastroesophageal reflux, there are no clear recommendations from scientific societies on which technique is superior and what criteria should be considered when indicating one or the other [37]. In this regard, following the SLEEVEPASS and SM-BOSS trials in 2018 (two mid-term RCTs comparing both techniques) [9, 10], Arterburn et al. [8] highlighted five different issues that could help shared decision making between surgical techniques for the individual patient: (1) the decision should be made jointly between the patient and medical team, (2) there are no differences in weight loss, (3) the presence of type 2 diabetes does not make RYGB preferable to SG, (4) RYGB is preferred in patients with gastroesophageal reflux disease, and (5) there are no differences in the rate of postsurgical reoperation. Thus, in accordance with the current evidence, we propose two modifications to Arterburn et al.’s [8] concepts. First, based on the BASALTO results, elevated levels of LDL cholesterol levels should be a new factor to consider in the choice of bariatric surgery technique. Second, the clear superiority of RYGB in terms of remission of elevated levels of LDL cholesterol found in the present study, as well as the higher remission rates of type 2 diabetes and hypertension observed in other RCTs and meta-analyses reported after 2018 [11, 38], means that the surgical techniques are not equivalent when deciding on the optimal procedure for each patient. Furthermore, this should be reflected in trending changes in RYGB indications in the coming years.

Moreover, both techniques were similar regarding changes in HDL cholesterol and triglyceride levels, with an overall improvement that has been previously outlined [9, 10, 14, 39, 40]. This emphasizes the need to report and treat high LDL cholesterol concentrations, in addition to other lipid disturbances, and not to use the general term ‘dyslipidemia’, as recommended by the ASMBS Clinical Issues Committee [41]. Using the term “dyslipidemia” to encompass triglyceride, HDL, and LDL abnormalities may obscure the specific effects observed on LDL cholesterol. In patients with atherogenic dyslipidemia and desirable LDL cholesterol concentration SG has the same potential to achieve dyslipidemia remission as bypass surgery. This helps to explain why the differences in dyslipidemia remission rates between the techniques reported by previous studies are lower than those detected for LDL cholesterol remission, the main driver of cardiovascular risk [40]..

The present study went beyond the conventional analysis of lipid profiles and evaluated changes in the composition and size of the different lipoprotein particles. After both surgeries, a similar reduction in VLDL particles was observed. However, the most remarkable finding was a decrease in the number of LDL particles after RYGB, including the smaller particles that are considered the most atherogenic. Moreover, this improvement was present early after surgery. These findings confirm those reported by Kjellmo et al. [42]., where reductions in both small and dense LDL particles as well as large LDL particles were observed after RYGB and biliopancreatic diversion. On the other hand, although the composition and number of LDL particles were not reduced after SG, an increase in their size was detected. Similar results were described by Genua et al. [43] in a study that mainly included patients who underwent SG. The only study that compared the qualitative lipoprotein changes between both surgeries did not find significant differences; [44] the small sample size (6 RYGB and 8 SG) and the inclusion of normolipidemic patients in that study could explain the lack of differences.

In addition, changes in glycoproteins and the main circulating cholesteryl esters were explored. Of note, species acylating fatty acids derived from de novo synthesis (palmitoleic and oleic acids) have been suggested to have a role in atherosclerosis [45] and the ensuing increasing cardiovascular risk [46–48]. Interestingly, significant changes were limited to intragroup differences in cholesteryl palmitoleate in the SG arm. However, changes at 12 months between groups were not significantly different.

Furthermore, the BASALTO study has aimed to delve deeper into the mechanisms by which RYGB is superior to SG in achieving LDL cholesterol remission. The differential effect may be due to the cholesterol absorption rate, which is decreased following RYGB and increased following SG. Reduced absorption after bypass can be explained by the surgical procedure consisting of a 150-cm antecolic Roux limb with a 25-mm circular pouch-jejunostomy and the exclusion of 50 cm of the proximal jejunum. This is consistent with the fact that the reduction in LDL cholesterol levels is already evident at 3 months post-RYBG and that techniques with a higher degree of malabsorption result in greater reductions in LDL cholesterol concentration [1]. On the other hand, the decrease in cholesterol synthesis observed with both techniques and the increased absorption after SG may be explained by the caloric restriction that occurs after surgery. This aligns with findings from studies reporting similar outcomes following hypocaloric diets in individuals with obesity [49]. Similar results on cholesterol absorption and synthesis markers were found by Pihlajamäki et al. [50], but in their study, the restrictive technique used was gastric banding.

The short-term follow-up period in our study highlights the need to assess the mid-term evolution of LDL cholesterol levels, particularly given that peak weight loss typically occurs between 12 and 24 months post-bariatric surgery. Our hypothesis posits that the observed differences in LDL cholesterol between the two surgical techniques are likely to persist for two reasons. Firstly, the findings of the present study regarding cholesterol absorption metabolites suggest that factors beyond weight reduction alone may influence lipid metabolism following bariatric surgery. Secondly, a retrospective study spanning over a 5-year period, conducted by our research group, consistently showed these trends [13]. 

The present study has several key strengths. It is the first randomized clinical trial powered for detecting a clinically significant difference in LDL cholesterol remission at 12 months. Furthermore, this study provides novel knowledge on the mechanisms involved in lipoprotein profile changes after bariatric surgery. However, certain limitations should be acknowledged. First, while the single-center design reduced variation in operative and perioperative procedures, it may have made the study results less generalizable. Second, patients with LDL cholesterol > 4.91 nmol/l, familial hypercholesterolemia or those in secondary cardiovascular prevention were not included in the present trial; therefore, the results cannot be extrapolated to the overall population with severe obesity and high LDL cholesterol concentrations. Third, these results cannot be extrapolated to patients over 60 years old or those with a BMI greater than 60 kg/m², populations in which bariatric surgery has been demonstrated to be effective. [51,52] Forth, this study was powered to detect differences in LDL cholesterol remission; thus, potential differences in subgroups of diabetic and non-diabetic patients, as well as secondary outcomes such as weight loss, other obesity complications, or cholesterol esters, might not have been detected. Additionally, it’s important to note that the clinical guidelines on dyslipidemia management during patient selection not fully align with current recommendations, potentially affecting the generalizability of our findings.

### Conclusion

In conclusion, RYGB was superior to SG in terms of high LDL cholesterol remission. Furthermore, RYGB also led to a greater improvement in other atherogenic lipoprotein fractions. Therefore, the presence of elevated levels of LDL cholesterol should be considered when determining the optimal bariatric surgery procedure for each patient.

### Electronic supplementary material

Below is the link to the electronic supplementary material.

Supplementary Material 1

## Data Availability

Individual participant data are not directly available but may be avail-able after study close upon reasonable request from the authors.

## Abbreviations

BMI: Body mass index
FAMEs: Fatty acid methyl esters
GC‒MS: Gas chromatography‒mass spectrometry
HbA1c: Glycated hemoglobin
LDL: Low-density lipoprotein
RCTs: Randomized controlled trials
RYGB: Roux-en-Y gastric bypass
SG: Sleeve gastrectomy
