# Supplementary material for: Short-term effects of gastric bypass versus sleeve gastrectomy on high LDL cholesterol: The BASALTO randomized clinical trial
Source: Cardiovasc Diabetol. 2024 Jun 15;23:205. doi: 10.1186/s12933-024-02296-x (PMC11180388; doi:10.1186/s12933-024-02296-x)
Supplement: Supplementary file 1 — Supplementary Material 1 [file 12933_2024_2296_MOESM1_ESM.pdf]

Supplemental Material

Supplemental Figure S1: Evolution of conventional lipid profile during follow-up with Roux-en-Y Gastric Bypass and Sleeve Gastrectomy.

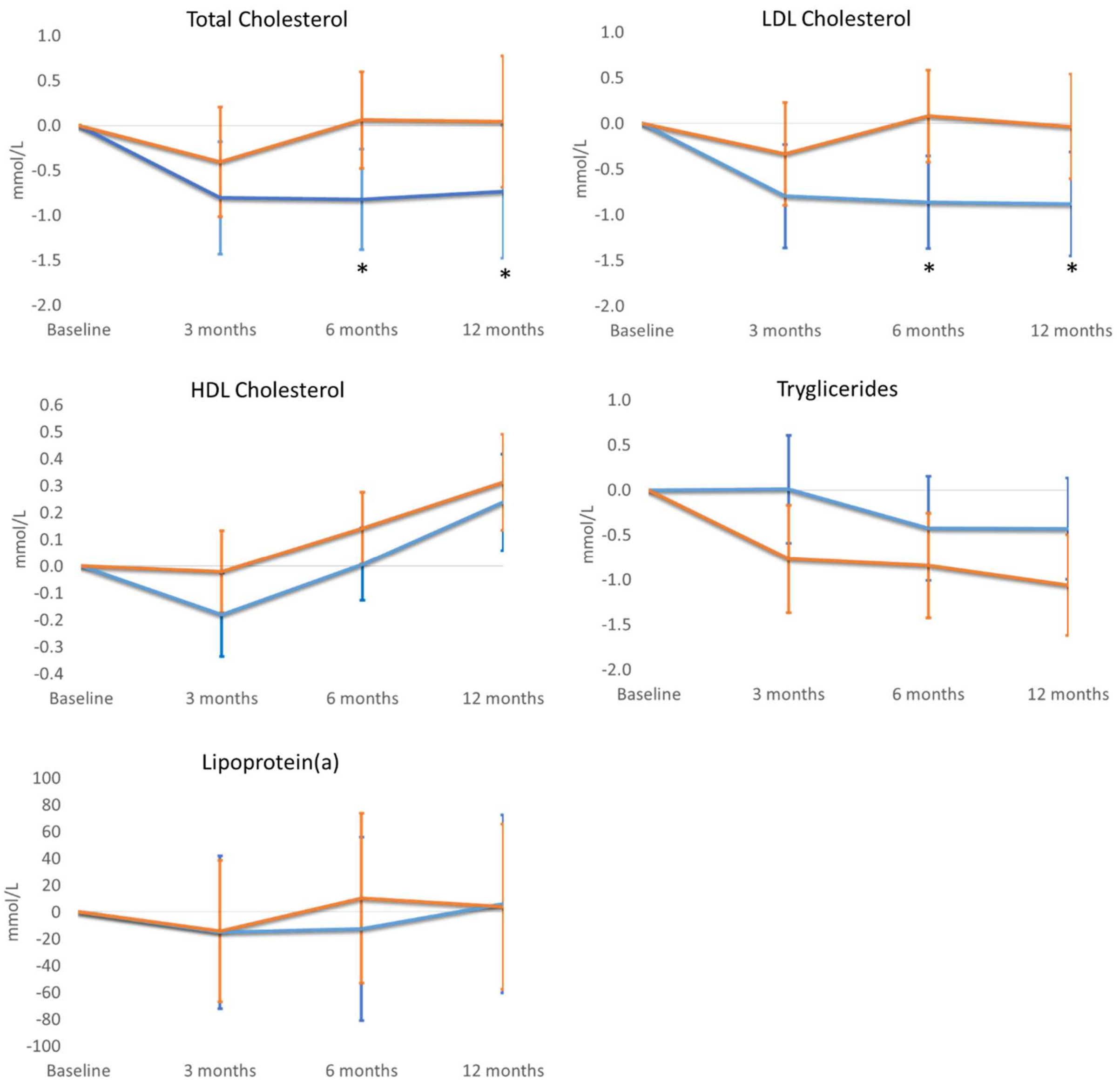

Negative values indicate a reduction, whereas positive values indicate an increase.

Data were expressed as means with 95% confidence interval.

*p <0.05. p value refers to the comparison between groups at each time interval. The changes in these parameters were analysed using ANOVA test for repeated measures ($p < 0.05$) adjusted for baseline triglycerides.

Supplemental Figure S2: Evolution of LDL cholesterol outcomes during follow-up with Roux-en-Y Gastric Bypass and Sleeve Gastrectomy.

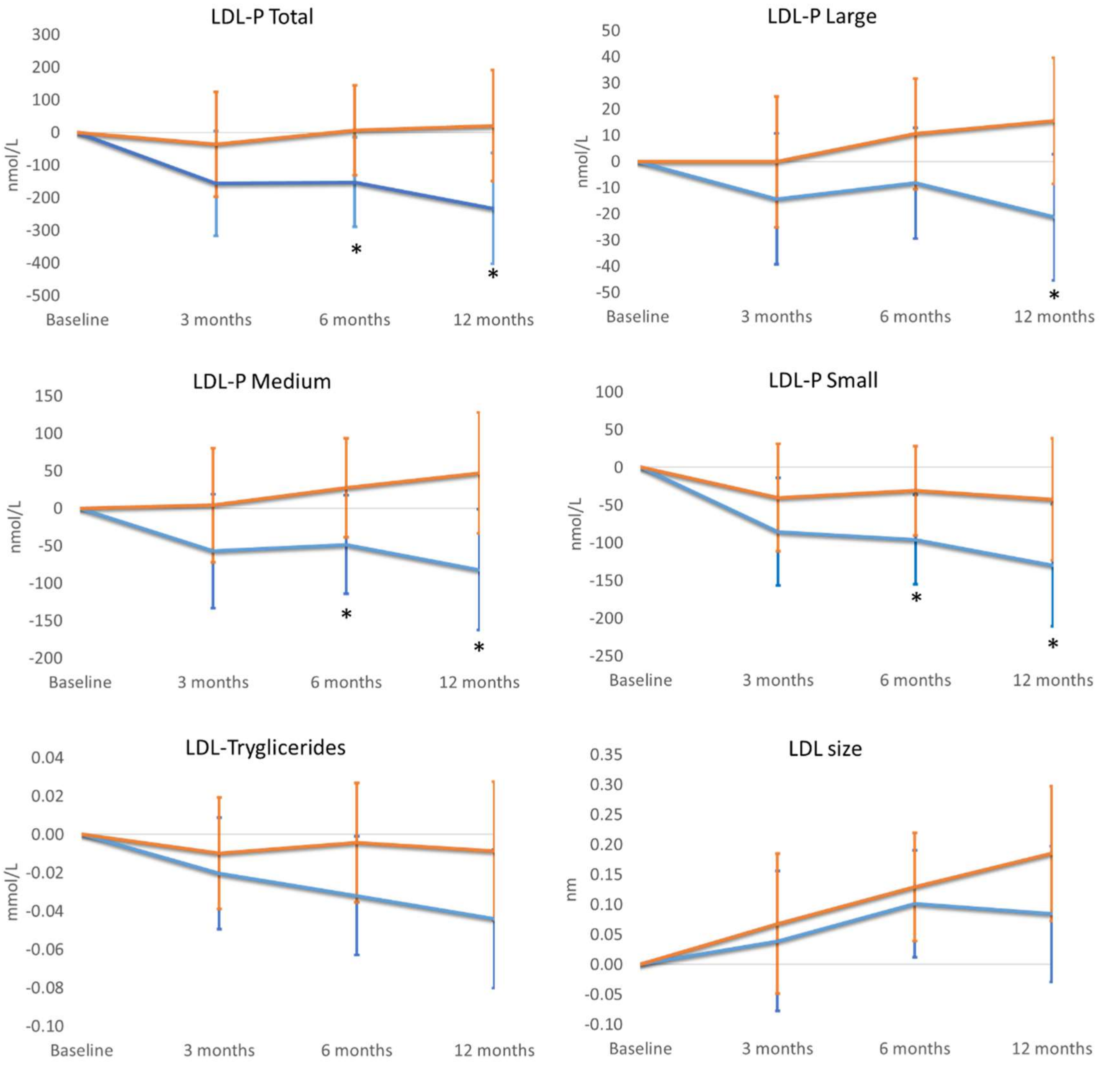

Negative values indicate a reduction, whereas positive values indicate an increase.

Data were expressed as means with 95% confidence interval.

*p <0.05. p value refers to the comparison between groups at each time interval. The changes in these parameters were analysed using ANOVA test for repeated measures ($p < 0.05$) adjusted for baseline triglycerides.

Supplemental Figure S3: Evolution of VLDL cholesterol outcomes during follow-up with Roux-en-Y Gastric Bypass and Sleeve Gastrectomy.

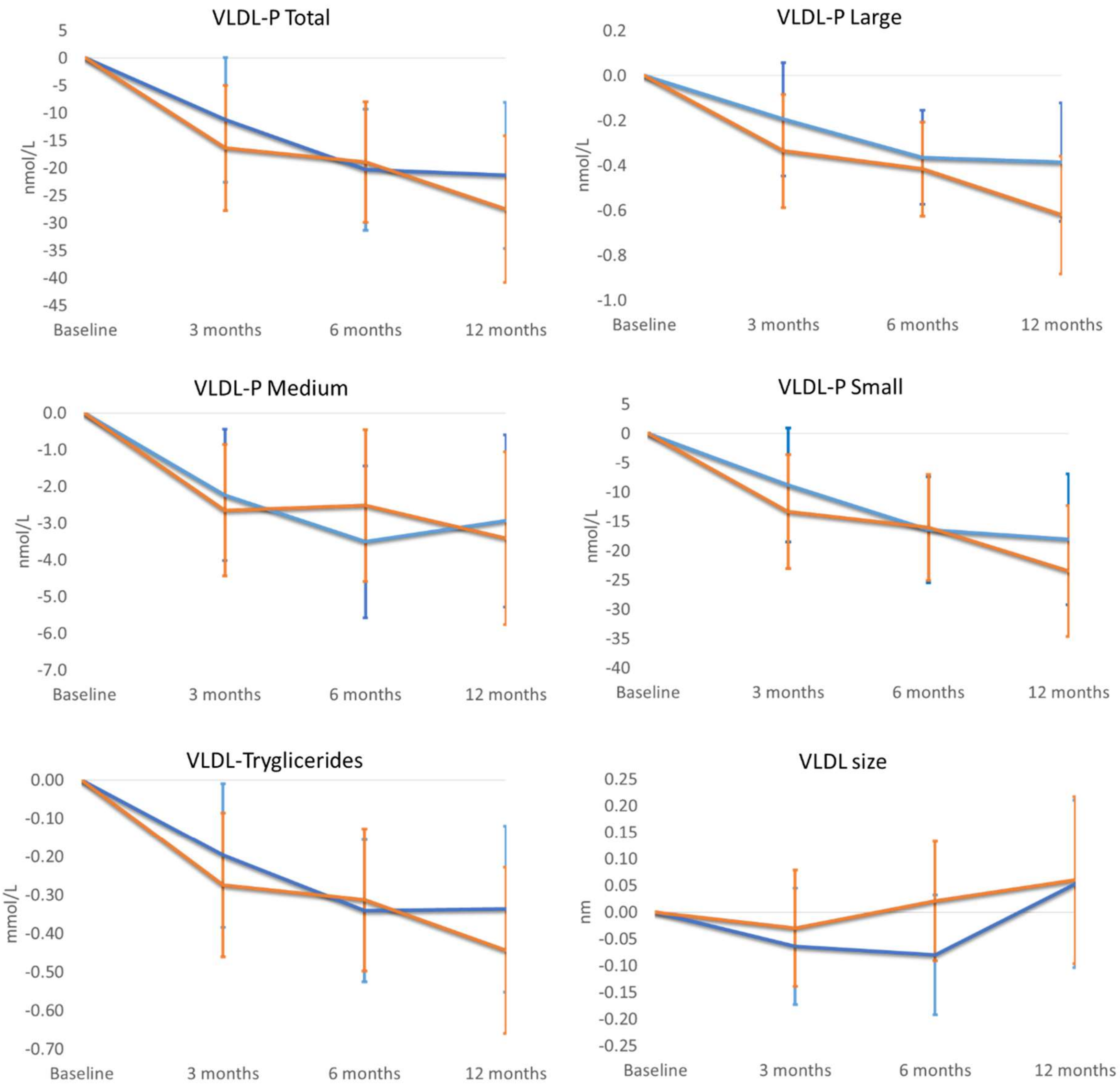

Negative values indicate a reduction, whereas positive values indicate an increase.

Data were expressed as means with 95% confidence interval.

*p <0.05. p value refers to the comparison between groups at each time interval. The changes in these parameters were analysed using ANOVA test for repeated measures ($p < 0.05$) adjusted for baseline triglycerides.

Supplemental Figure S4: Evolution of HDL cholesterol outcomes during follow-up with Roux-en-Y Gastric Bypass and Sleeve Gastrectomy.

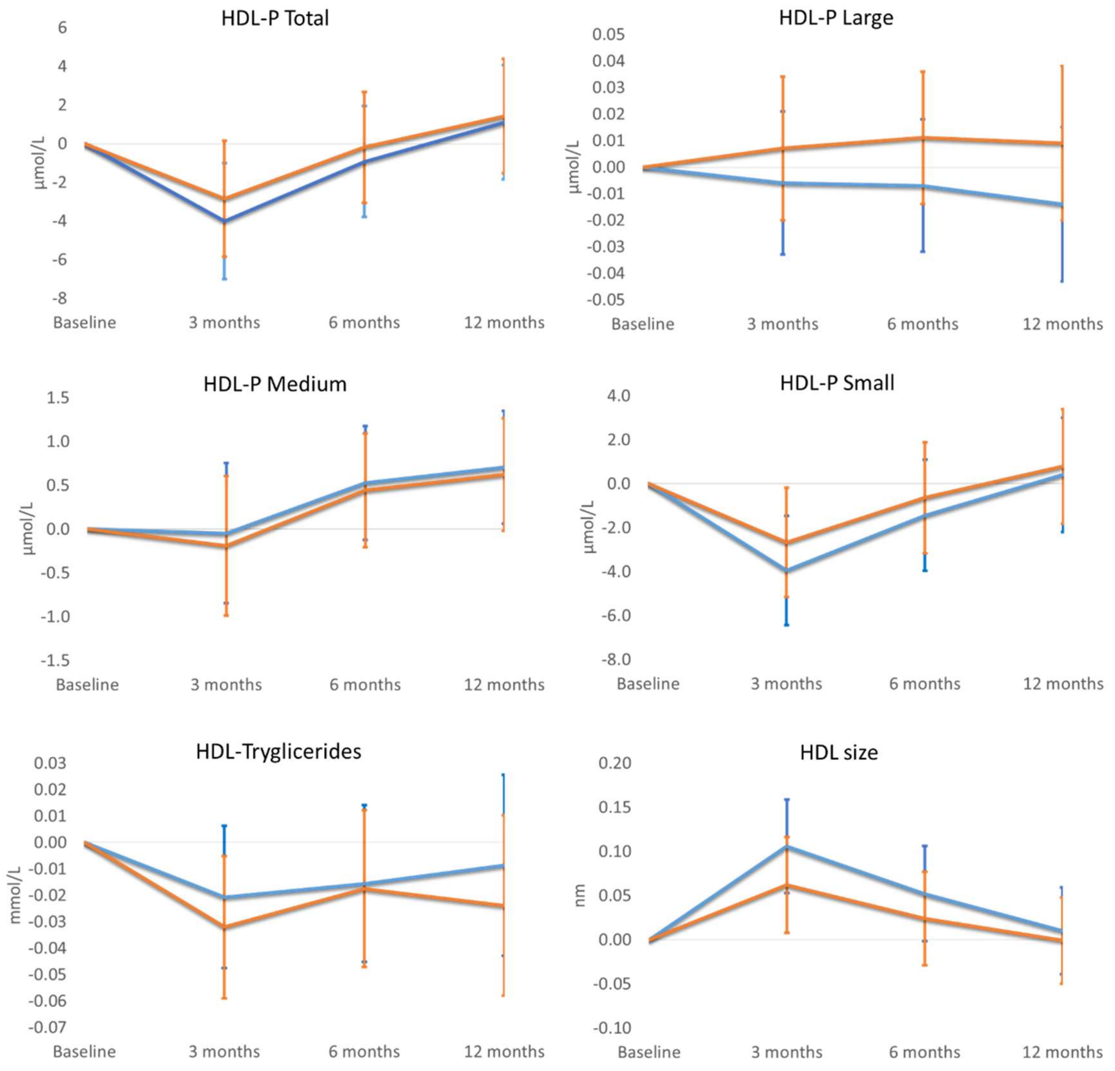

Negative values indicate a reduction, whereas positive values indicate an increase.

Data were expressed as means with 95% confidence interval.

*p <0.05. p value refers to the comparison between groups at each time interval. The changes in these parameters were analysed using ANOVA test for repeated measures ($p < 0.05$) adjusted for baseline triglycerides.

Supplemental Figure S5: Evolution of Glycoproteins during follow-up with Roux-en-Y Gastric Bypass and Sleeve Gastrectomy.

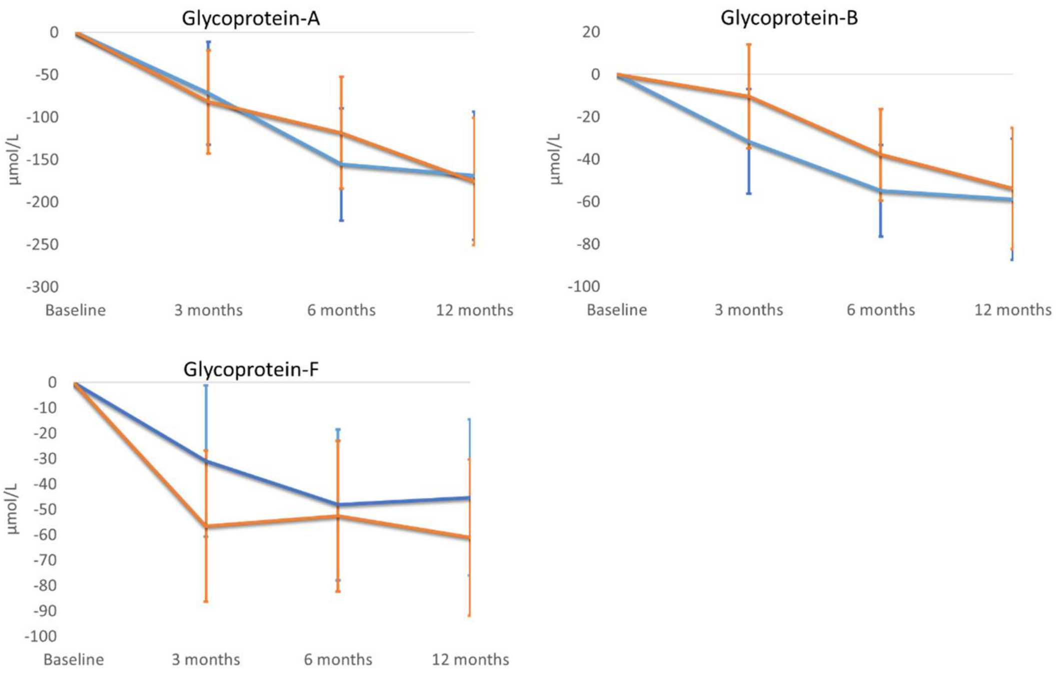

Negative values indicate a reduction, whereas positive values indicate an increase.

Data were expressed as means with 95% confidence interval.

*p <0.05. p value refers to the comparison between groups at each time interval. The changes in these parameters were analysed using ANOVA test for repeated measures ($p < 0.05$) adjusted for baseline triglycerides.

Supplemental Figure S6: Evolution of Cholesterol esters during follow-up with Roux-en-Y Gastric Bypass and Sleeve Gastrectomy.

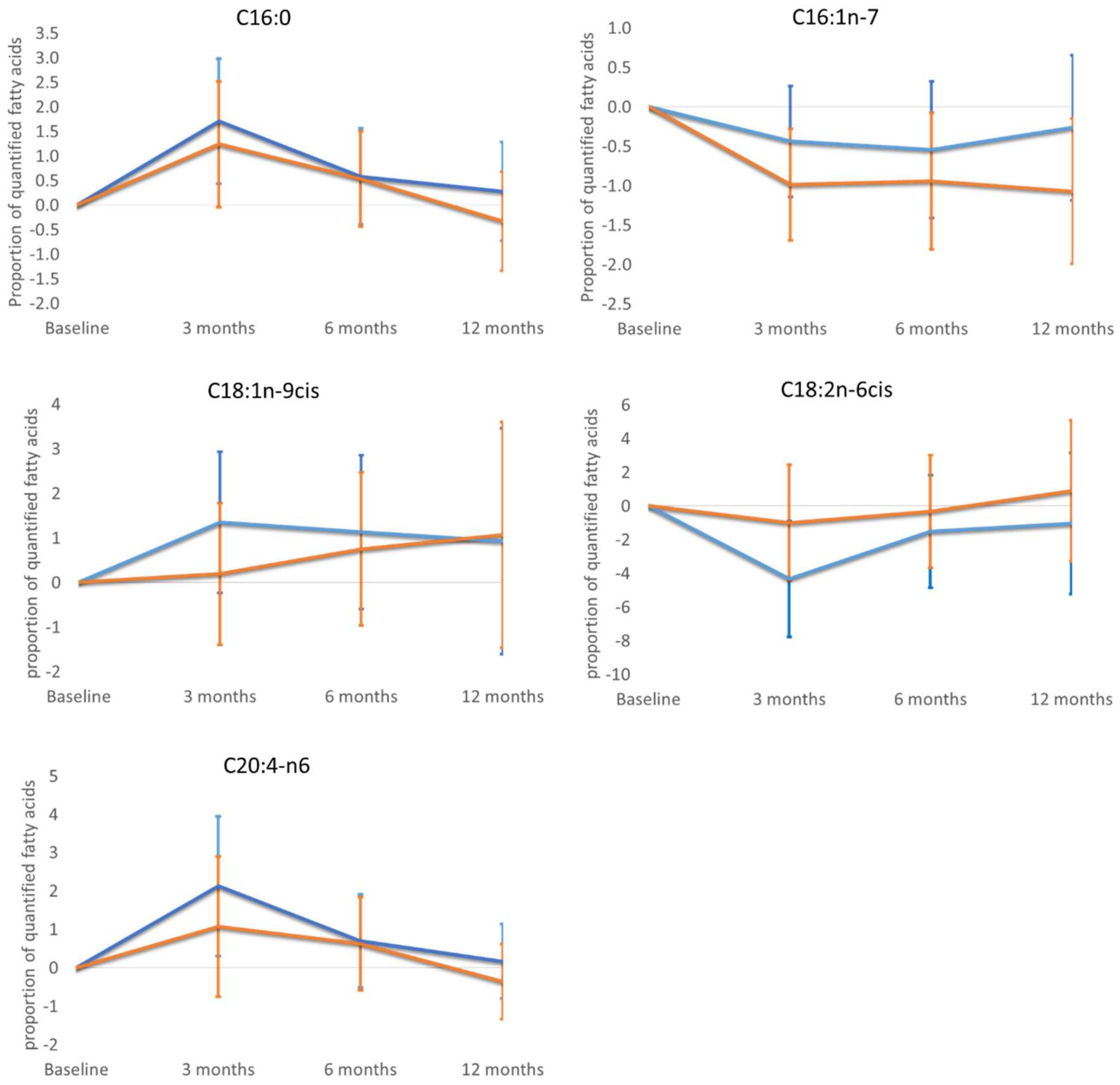

Negative values indicate a reduction, whereas positive values indicate an increase.

Data were expressed as means with 95% confidence interval.

*p <0.05. p value refers to the comparison between groups at each time interval. The changes in these parameters were analysed using ANOVA test for repeated measures ($p < 0.05$) adjusted for baseline triglycerides.

Supplemental Figure S7: Evolution of Cholesterol absorption and synthesis biomarkers during follow-up with Roux-en-Y Gastric Bypass and Sleeve Gastrectomy.

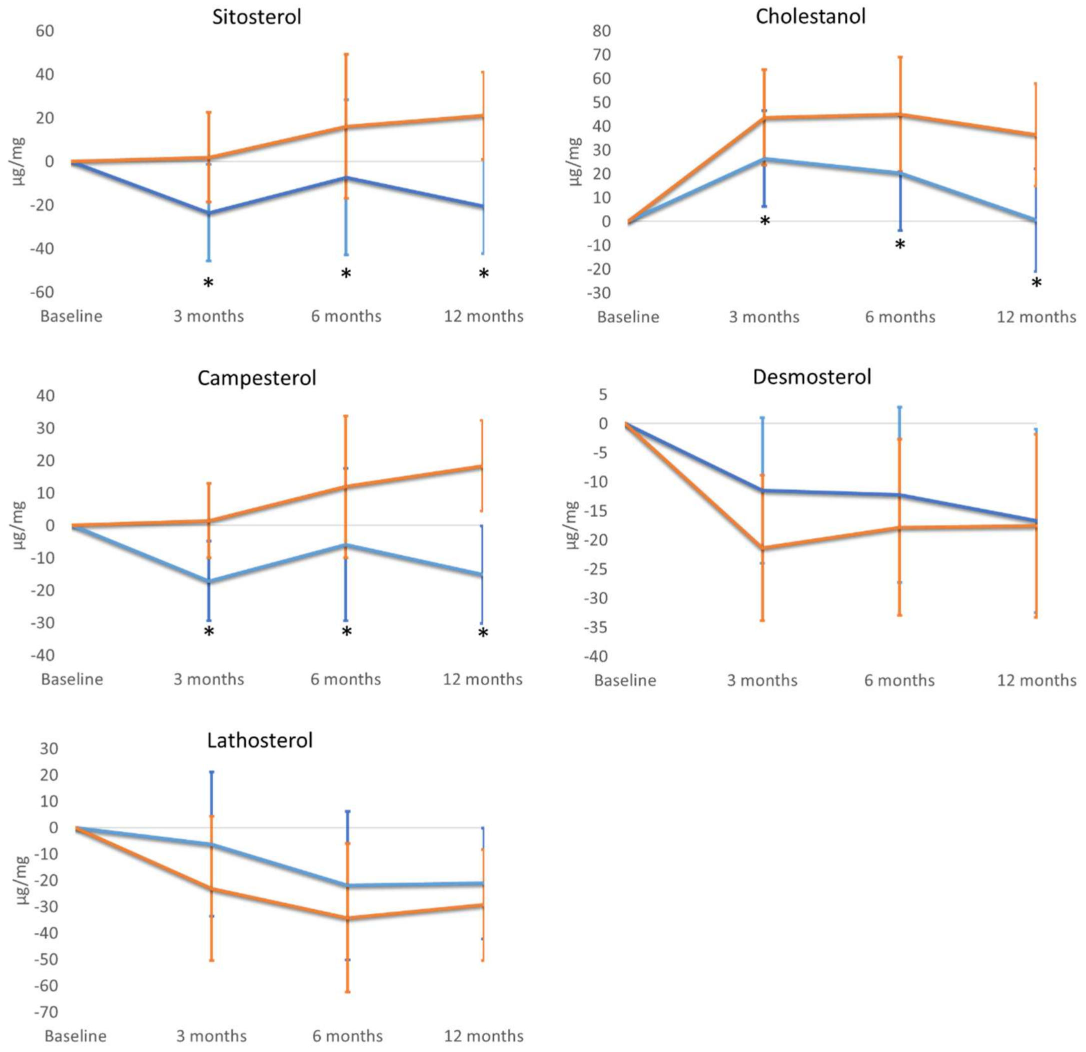

Negative values indicate a reduction, whereas positive values indicate an increase.

Data were expressed as means with 95% confidence interval.

*$p < 0.05$. p value refers to the comparison between groups at each time interval. The changes in these parameters were analysed using ANOVA test for repeated measures ($p < 0.05$) adjusted for baseline triglycerides.
